# Dietary Attitude of Adults with Type 2 Diabetes Mellitus in the Kingdom of Saudi Arabia: A Cross-Sectional Study

**DOI:** 10.3390/medicina56020091

**Published:** 2020-02-24

**Authors:** Waqas Sami, Khalid M Alabdulwahhab, Mohd Rashid Ab Hamid, Tariq A. Alasbali, Fahd Al Alwadani, Mohammad Shakil Ahmad

**Affiliations:** 1 Department of Community Medicine and Public Health, College of Medicine, Majmaah University, Al-Majmaah 11952, Saudi Arabia; m.shakil@mu.edu.sa; 2 Department of Ophthalmology, College of Medicine, Majmaah University, Al-Majmaah 11952, Saudi Arabia; k.alabdulwahhab@mu.edu.sa; 3 Centre for Mathematical Sciences, Universiti Malaysia Pahang, Lebuhraya Tun Razak, Gambang, Kuantan, Pahang 26300, Malaysia; rashid@ump.edu.my; 4 Department of Ophthalmology, College of Medicine, Al-Imam Mohammad Ibn Saud Islamic University, Riyadh 7544, Saudi Arabia; taalasbali@imamu.edu.sa; 5 Department of Ophthalmology, College of Medicine, King Faisal University, Hofuf 31982, Saudi Arabia; dr_wadani@yahoo.com

**Keywords:** dietary attitude, type 2 diabetes mellitus, diabetes self-management, empowerment approach, dietary behavior

## Abstract

*Background and Objectives:* There is a paucity of literature on the dietary attitude (DA) of patients with type 2 diabetes in the Kingdom of Saudi Arabia (KSA). Although the prevalence of diabetes mellitus (DM) is high in Gulf countries, there remains a lack of understanding of the importance of dietary behavior in diabetes management among patients. Understanding the behavior of patients with diabetes towards the disease requires knowledge of their DA. Therefore, this study aimed to assess and evaluate the DA of type 2 diabetes patients, and it is the first of its kind in the KSA. *Material and Methods:* An analytical cross-sectional study was conducted among 350 patients with type 2 diabetes. A self-administered DA questionnaire was used to collect the data. Psychometric properties of the questionnaire were assessed by face validity, content validity, exploratory factor analysis, and internal consistency reliability. The data were collected using a systematic random sampling technique. *Results:* The overall DA of the patients was inappropriate (*p* = 0.014). Patients had an inappropriate DA towards food selection (*p* = 0.003), healthy choices (*p* = 0.005), food restraint (*p* < 0.001), health impact (*p* < 0.001), and food categorization (*p* = 0.033). A poor DA was also observed in relation to the consumption of red meat (*p* <0.001), rice (*p* < 0.001), soup and sauces (*p* = 0.040), dairy products (*p* = 0.015), and junk food (*p* < 0.001). *Conclusions:* It is highly recommended that patients with diabetes receive counseling with an empowerment approach, as this can bring about changes in their dietary behavior, which is deeply rooted in their daily routine. Healthcare providers should also be well-informed about patients’ attitudes and beliefs towards diabetes to design tailored educational and salutary programs for this specific community. Diabetes self-management educational programs should also be provided on a regular basis with a special emphasis on diet and its related components.

## 1. Introduction

Dietary attitude (DA) is defined as beliefs, thoughts, and feelings about, behaviors toward, and relationships with food. It can influence people’s food choices and their health status [1]. Different DAs affect human health in noncommunicable diseases and play a great role in determining cultural differences [2,3]. Local and international literature assessing the DA of patients with type 2 diabetes is very scarce. However, some studies have shown that assessing patients’ DA may have a considerable benefit for treatment compliance and decreases the occurrence rate of complications as well [4]. Unhealthy eating habits, failure to follow a strict diet plan, and physical inactivity are the leading causes of complications among patients with type 2 diabetes mellitus (T2DM) [5]. A study conducted in Egypt reported that the attitude of patients towards food, compliance with treatment, food control with and without drug use, and foot care was inadequate [6]. Another study indicated that only one-third of diabetic patients were aware of the importance of diet planning and limiting cholesterol intake to prevent cardiovascular disease (CVD) [7]. A study conducted in the Kingdom of Saudi Arabia (KSA) reported that diabetic patients do not regard the advice given by their physicians regularly for diet planning, diet modification, and exercise [8]. There is a need for patients with diabetes to develop a positive attitude towards diet that would help improve glycemic control, and eventually increase their health-related quality of life [9].

Although the prevalence of diabetes mellitus (DM) is high in Gulf countries (Kuwait, Qatar, Bahrain, United Arab Emirates, and Oman), there remains a lack of understanding of the importance of dietary behavior in diabetes management among patients [10]. Understanding the behavior of patients with diabetes towards the disease requires knowledge of their DA. Therefore, this study aimed to assess and evaluate the DA of type 2 diabetes patients. Since this is the first study in the KSA to focus on this issue, the results can therefore serve as a baseline for similar studies conducted in the KSA and in the neighboring Gulf countries.

## 2. Materials and Methods

The study was performed using an analytical cross-sectional design. Data were collected from the patients visiting the Primary Healthcare Centers (PHCs) in Majmaah City, KSA from February to April 2017. A systematic random sampling technique was used for the selection of patients based on the inclusion criteria, which were: clinically diagnosed cases of type 2 diabetes mellitus of either gender and in the age range of 35–55 years. The DM prevalence value of 23.7% [11] was used for sample size calculation, and the values were placed in the level of precision formula that yielded a sample size of 278. To compensate for potential missing observations/patients withdrawing from the study, the sample size was increased to 350. Each patient’s consent was obtained prior to data collection. This research was approved by the ethical review committee of Majmaah University, KSA vides reference number: MURECApril.02/COM-2016.

The dietary attitude questionnaire (DAQ) was prepared following a thorough review of the literature and based on meetings with local experts to determine the pattern of questions suitable for assessing and evaluating the DA of patients with type 2 diabetes. The self-administered valid and reliable questionnaire was divided into three sections (Section A, B, and C). We have discussed the psychometric properties (face validity, content validity, exploratory factor analysis (EFA), and reliability) of the DAQ in a separate article [12]. The internal consistency reliability of the DAQ was excellent (Cronbach Alpha = 0.841). Based on the pilot study results of the EFA, the five factors were labelled as “food selection”, “health impact”, “healthy choices”, “food restraint”, and “food categorization” [12].

Section A contained questions related to demographic characteristics. Section B was comprised of 16 questions that assessed patients’ general DA towards food. All of the questions were measured on a seven-point Likert scale (strongly agree, agree, somewhat agree, neutral, disagree, somewhat disagree, and strongly disagree). The DA was further classified as positive and negative based on mean values. Values at or above the mean were classified as having a positive DA, and values below the mean were referred to as having a negative DA [13,14]. Section C was also comprised of 16 questions: The first 15 questions assessed patients’ DA towards specific food items with categories (“not” eating this food is healthy and necessary, eating this food “occasionally” is healthy and necessary, and eating this food “often” is healthy and necessary), and the last question was about “opinion regarding healthy diet” with the options “yes” and “no”.

The data were entered and analyzed using IBM SPSS version 25 (IBM Corp., Armonk, N.Y., USA). Normality of the quantitative variables was assessed through a One-Sample Kolmogorov–Smirnov (KS) test. A univariate method (z-score) was used for the detection of outliers. Qualitative variables are expressed as frequencies and percentages, while a median and quartiles (25th–75th) are given for non-normally distributed variables. A one-sample non-parametric chi-squared test was used to assess the significance of overall and subgroup positive and negative DA. Pearson’s chi-squared test was applied to compare the overall positive and negative DA between gender, body mass index (BMI), education status, and marital status. Binary logistic regression with the backward conditional approach was used to predict the set of variables assessing the DA of patients towards specific food items. The odds ratios were further converted into probabilities by using the equation (ŷ = odds/1 + odds). The statistical significance value was set at *p* < 0.05.

## 3. Results

### 3.1. Demographic Characteristics of Patients—Section A

The data were collected from 350 patients with a median age of 45 years (range: 40–51 years). The results presented in Table 1 show that there were more male patients (*n* = 202; 57.7%) than female patients (*n* = 148; 42.3%). More than 90% of the patients were married. A majority of patients had received a secondary education (*n* = 200; 57.14%), while some were illiterate (*n* = 69; 19.7%), and others were graduates and postgraduates (*n* = 81; 23.14%). A majority of patients in the study were overweight (*n* = 167; 47.7%), some were obese (*n* = 115; 32.9%), some had a normal weight (*n* = 56; 16%), and others were underweight (*n* = 12; 3.4%). A significant association was observed between the overall DA of patients and their educational status (*p* = 0.034). However, the overall DA was not significantly associated with gender (*p* = 0.142), marital status (*p* = 0.413), or BMI (*p* = 0.666). The frequency, percentage, and ranked mean score for each item are presented in Table 2.

### 3.2. Patients’ General Dietary Attitude Towards Food—Section B

No outlier problem was detected in the overall DA score variable as z-score values (−2.30–2.22) were less than the absolute value of 4. The mean DA score of 16 items was 3.94 + 0.87. Based on the mean score, the DA was categorized into having a positive attitude and having a negative attitude. There was a majority of patients with a negative DA (*n* = 198; 56.6%) compared with those with a positive DA (*n* = 152; 43.4%). The result of the one-sample chi-squared test showed that the overall DA of patients with type 2 diabetes was inappropriate (χ^2^ = 6.04 (1), *p* = 0.014). The positive and negative attitude when compared within the subgroups (identified by EFA) showed that the patients also had an inappropriate DA towards food selection (*p* = 0.003), healthy choices (*p* = 0.005), food restraint (*p <* 0.001), health impact (*p* < 0.001), and food categorization (*p* = 0.033). These results are presented in Table 3.

### 3.3. Patients’ General Dietary Attitude Towards Food—Section B

Backward elimination with the conditional approach retained six items in the final model. The values of model chi-squared and Hosmer–Lemeshow tests were 81.80 (*p* < 0.001) and 20.02 (*p* < 0.001), respectively, which showed that the fitted model was appropriate at the 95% confidence interval (CI). Overall, the model correctly classified 71.4% of patients. The odds ratio for red meat was 2.43 (*p* < 0.001). Converting the odds ratio into a probability showed that the consumption of red meat was 70.84% greater in patients who said “yes” they are eating a healthy diet. Dairy products had an odds ratio of 1.408 (*p* = 0.015), which showed that the consumption of dairy products was 58.38% greater in patients who said “yes” they are eating a healthy diet. The odds ratio for rice was 3.472 (*p* < 0.001). The probability results showed that consumption of rice was 77.63% greater in patients who said “yes” they are eating a healthy diet. Junk food had an odds ratio of 2.347 (*p* < 0.001), showing that the consumption of junk food was 70.12% greater in patients who said “yes” they are eating a healthy diet. The odds ratio for soups and sauces was 1.383 (*p* = 0.040). The probability results showed that the consumption of soups and sauces was 58.03% greater in patients who said “yes” they are eating a healthy diet. Fruits had an odds ratio of 1.416 (*p* = 0.024). Converting the odds ratio into a probability showed that the consumption of fruits was 58.60% greater in patients who said “yes” they are eating a healthy diet. However, for foods such as white meat, bakery products, cereals, sweets and snacks, drinks, vegetables, boiled or grilled meals, olive oil, and canned food, there was no statistical significance (*p* > 0.05). These results are presented in Table 4.

## 4. Discussion

Our study showed that patients with type 2 diabetes had an overall inappropriate DA. Subgroup analysis also showed an inappropriate DA of patients towards food selection, health impact of food, healthy choices, food restraint, and food categorization. In addition, the patients had a poor DA towards the consumption of red meat, rice, soup and sauces, dairy products, and junk food. The results of our study also showed that for the majority of patients, food selection and health impact of food were not important, and this is consistent with the findings of a study conducted in Egypt [6]. This may be because of deeply rooted cultural beliefs and values, which may pose a difficulty for patients’ adherence to food selection and consumption of foods having a health impact. The role of cultural attitudes and behaviors towards food in the management of diabetes cannot be neglected [15]. This is consistent with our study results, as the attitude of patients with diabetes towards food is influenced by a strong cultural attitude. Most of them stated that the selection of food, its health impact, healthy choices, food restriction, and food categorization are not important to them. The Saudi cultural barrier factor towards food selection and its consumption and health impact has also been supported by a local study [16]. In our study, a majority of the patients stated that they do not like to eat diet food, nor do they like to stay away from foods that contain sugar. Moreover, only one-fifth of the patients indicated that they feel guilty after eating oily foods. These findings are supported by research conducted by Buttar et al. [17].

A study conducted by Ntaate [18] among patients with type 2 diabetes from Uganda reported a positive DA (82%) towards diet. In contrast, in our study, the patients not only had an overall inappropriate DA, but also an inappropriate DA towards the consumption of red meat, rice, soup and sauces, dairy products, and junk food. Most of the patients in our study were unaware of the caloric content in the food they were consuming. This can be attributed to their literacy level; in our study, 57.2% of the patients had received a primary and secondary education, while approximately 20% were illiterate. This fact is supported by studies that also stated that literacy is an important influential factor, because patients with low literacy have difficulty reading food labels and estimating potion sizes [19,20,21].

Therefore, to achieve the DA goals, a patient empowerment approach should be used. Since an empowerment approach is a social phenomenon, when a patient is empowered with necessary knowledge about lifestyle modification, outcomes of disease if not controlled, etc., he/she shows a more responsible attitude with better self-efficacy towards diabetes care [22,23]. The empowerment approach in dealing with type 2 diabetes is highly recommendable because it brings about changes in the behavior of the patient that is deeply rooted in their daily routine. Healthcare providers should be well-informed about patient attitudes and beliefs towards diabetes to design tailored educational and salutary programs for a specific community [24].

Imparting nutritional education is a perilous component of diabetes care, especially for the self-management of the disease. Thus, for better diabetes care, patients should be referred to dietitians who should assess their attitude towards food in general, and towards various foods such as meat, rice, junk food, etc., and suggest tailored dietary self-management strategies. To facilitate behavioral dietary changes, this assessment should be individualized and patient-centered, and it must be based on a patient’s cultural beliefs, norms, psychosocial status, and literacy, as these factors have been identified as a barrier to reaching nutritional therapy goals [25]. Along with these efforts, the authorities in the Kingdom of Saudi Arabia should provide diabetes self-management educational programs on a regular basis, with special emphasis on diet and its related components. Such educational programs have been found to have an encouraging impact on patient behaviors. However, to achieve a long-term positive effect on behavior modification, sustained reinforcement is needed, which can be achieved using a patient empowerment approach [26].

There are some limitations to this study. The research design was cross-sectional, which itself has methodological limitations, so it cannot be used to analyze behavior over a period of time. The study was conducted in the central region of the KSA, and although the eating habits do not vary much within the eastern, southern and northern regions of the KSA, there is still a need for a national DA assessment program. Another limitation is that we were unable to compare the self-prepared DAQ with the gold standard; doing so might have helped us to study the DA of the patients with diabetes in more detail to devise strategies for better patient care. Nonetheless, the study provided important points: The results can be generalized as we used a systematic random sampling technique for the selection of patients, the DA questionnaire was reviewed by experts in the field, it successfully passed the psychometric analysis, and we can say that it is a valid and reliable questionnaire for assessing and evaluating the DA of patients with type 2 diabetes. This is the first study conducted in the KSA related to assessing and evaluating the DA of patients with type 2 diabetes. Therefore, the results can serve as a baseline for similar studies conducted in the KSA and references can be extended to the neighboring Gulf Cooperation Council (GCC) countries.

## 5. Conclusions

Patients with type 2 diabetes had an overall inappropriate dietary attitude. It is highly recommended that these patients be counseled with an empowerment approach as it can bring about changes in their dietary behavior that is deeply rooted in their daily routine. Healthcare providers should also be well-informed about patients’ attitudes and beliefs towards diabetes to plan tailored educational and salutary programs for this specific community. Diabetes self-management educational programs should also be provided on a regular basis with a special emphasis on diet and its related components.

## Figures and Tables

**Table 1:** Sociodemographic Characteristics.

	***n*(%)**		***n*(%)**
**Gender**		**Education Status**	
		Illiterate	69 (19.7)
Male	202 (57.7)	Primary	115 (32.9)
		Secondary	85 (24.3)
		Graduates	51 (14.6)
Female	148 (42.3)	Postgraduates	30 (8.6)
**Marital Status**		**BMI**	
Married	322 (92.0)	Underweight	12 (3.4)
Single	11 (3.1)	Normal weight	56 (16.0)
Widow	06 (1.7)	Overweight	167 (47.7)
Divorced/Separated	11 (3.1)	Obese	115 (32.9)

BMI, Body mass index.

**Table 2:** General dietary attitude of patients with type 2 diabetes based on ranking analysis.

Items	Strongly Disagree	Somewhat Disagree	Disagree	Neutral	Agree	Somewhat Agree	Strongly Agree	Mean Score
	*n* (%)	*n* (%)	*n* (%)	*n* (%)	*n* (%)	*n* (%)	*n* (%)	Mean ± SD
It is important that the food you eat keeps you healthy and energetic	6 (1.7)	32 (9.1)	61 (17.4)	24 (6.9)	109 (31.1)	75 (21.4)	73 (12.3)	4.70 ± 1.77
You are aware of the energetic (caloric) content in the food that you eat	0 (0.0)	32 (9.1)	81 (23.1)	45 (12.9)	68 (19.4)	75 (21.4)	49 (14.0)	4.63 ± 1.58
It is important that the food that you eat contains vitamin and minerals	13 (3.7)	53 (15.1)	37 (10.6)	71 (20.3)	99 (28.3)	47 (13.4)	30 (8.6)	4.29 ± 1.60
You feel guilty after eating oily foods	16 (4.6)	36 (10.3)	84 (24.0)	60 (17.1)	77 (22.0)	47 (13.4)	30 (8.6)	4.16 ± 1.60
The healthiness of food has little impact on your food choices	0 (0.0)	38 (10.9)	93 (26.6)	59 (16.9)	97 (27.7)	63 (18.0)	0 (0.0)	4.15 ± 1.29
You generally feel comfortable after eating sweets	0 (0.0)	71 (20.3)	42 (12.0)	108 (30.9)	25 (7.1)	104 (29.7)	0 (0.0)	4.14 ± 1.47
You give too much time and thought to food selection	35 (10.0)	43 (12.3)	37 (10.6)	68 (19.4)	90 (25.7)	47 (13.4)	30 (8.6)	4.13 ± 1.74
It is important that the food you eat helps you control your weight	23 (6.6)	67 (19.1)	61 (17.4)	28 (8.0)	72 (20.6)	83 (23.7)	16 (4.6)	4.06 ± 1.76
You enjoy trying new, rich, nutritious food	0 (0.0)	37 (10.6)	87 (24.9)	106 (30.3)	74 (21.1)	46 (13.1)	0 (0.0)	4.01 ± 1.18
You like to consume food cooked in olive oil (virgin, extra, etc.)	38 (10.9)	45 (12.9)	62 (17.7)	48 (13.7)	115 (32.9)	13 (3.7)	29 (8.3)	3.89 ± 1.69
You can show self-control around food	26 (7.4)	62 (17.7)	62 (17.7)	42 (12.0)	122 (34.9)	21 (6.0)	15 (4.3)	3.84 ± 1.58
You try to stay away from foods such as bread, potato, and rice	0 (0.0)	106 (30.3)	102 (29.1)	12 (3.4)	36 (10.3)	94 (26.9)	0 (0.0)	3.74 ± 1.61
You eat what you like to eat and do not worry about the healthiness of food	50 (14.3)	58 (16.6)	57 (16.3)	42 (12.0)	105 (30.0)	38 (12.9)	0 (0.0)	3.59 ± 1.64
You stay away from foods that contain sugar	41 (11.7)	103 (29.1)	37 (10.6)	56 (16.0)	52 (14.9)	32 (9.1)	30 (8.6)	3.55 ± 1.84
Do you think that eating healthy food influences the outcomes of DM?	50 (14.3)	58 (16.6)	71 (20.3)	69 (19.7)	57 (16.3)	45 (12.9)	0 (0.0)	3.46 ± 1.59
You like to eat diet food	74 (21.1)	62 (17.7)	62 (17.7)	30 (8.6)	97 (27.7)	25 (7.1)	0 (0.0)	3.25 ± 1.67

DM, Diabetes mellitus; SD, Standard deviation.

**Table 3:** Comparison of Positive and Negative Dietary Attitude in Subgroups identified by exploratory factor analysis (EFA).

Food Selection	Health Impact	Healthy Choices	Food Restraint	Food Categorization
It is important that the food you eat contains vitamin and minerals	It is important that the food you eat keeps you healthy and energetic	You like to eat diet food	You eat what you like to eat and do not worry about the healthiness of food	The healthiness of food has little impact on your food choices
You stay away from foods that contain sugar	It is important that the food you eat helps you control your weight	You like to consume food cooked in olive oil (virgin, extra, etc.)	You can show self-control around food	You try to stay away from foods such as bread, potato, and rice
You give too much time and thought to food selection	You are aware of the energetic (caloric) content in the food that you eat	Do you think that eating healthy food has an effect on the outcomes of DM?	-	You enjoy trying new, rich, nutritious food
You feel guilty after eating oily foods	You generally feel comfortable after eating sweets	-	-	-
PDA = 147 (42.0%)	PDA = 149 (42.6%)	PDA = 140 (40%)	PDA = 126 (36%)	PDA = 155 (44.3)
NDA = 203 (58.0%)	NDA = 201 (57.4%)	NDA = 210 (60%)	NDA = 224 (64%)	NDA = 195 (55.7)
χ^2^ = 8.98, *p* = 0.003 *	χ^2^ = 7.72, *p* = 0.005 *	χ^2^ = 14.0, *p* < 0.001 *	χ^2^ = 27.4, *p* < 0.001 *	χ^2^ = 4.57, *p* = 0.033 *

DM, Diabetes mellitus; PDA, Positive Dietary Attitude; NDA, Negative Dietary Attitude; * statistically significant at the 5% level of significance.

**Table 4:** Binary Logistic Regression Analysis using the Backward Conditional Approach for the Dietary Attitude of Patients with Type 2 Diabetes towards Specific Food Items.

Food Item	β	Wald	*p*-Value	Adjusted Odds Ratio	95% CI for Odds	
					Lower	Upper
**White Meat**	0.098	0.345	0.557 †	0.907	0.654	1.257
**Red Meat**	0.888	0.175	0.000 *	2.430	1.726	3.422
**Dairy Products**	0.342	0.140	0.015 *	1.408	1.070	1.853
**Bakery Products**	0.136	0.611	0.434 †	0.873	0.662	1.227
**Rice**	1.245	0.209	0.000 *	3.472	2.303	5.233
**Cereals**	0.032	0.040	0.842 †	0.958	0.705	1.330
**Junk Food**	0.853	0.189	0.000 *	2.347	1.621	3.399
**Soups and Sauces**	0.324	0.158	0.040 *	1.383	1.015	1.884
**Sweets and Snacks**	0.060	0.136	0.712 †	1.062	0.772	1.460
**Drinks**	0.303	2.769	0.096 †	0.739	0.517	1.055
**Fruits**	0.348	0.154	0.024 *	1.416	1.047	1.914
**Vegetables**	0.074	0.180	0.671 †	1.077	0.765	1.515
**Boiled or Grilled Meals**	0.015	0.009	0.924 †	0.985	0.719	1.349
**Olive Oil**	0.079	0.210	0.647 †	0.924	0.660	1.295
**Canned Food**	0.223	1.696	0.193 †	0.800	0.573	1.119

* Significant at the 5% level of significance; † non-significant variables.
